# MEK inhibitor PD0325901 and vitamin C synergistically induce hypomethylation of mouse embryonic stem cells

**DOI:** 10.18632/oncotarget.9452

**Published:** 2016-05-18

**Authors:** Cuiping Li, Baodong Liu, Shangwei Zhong, Hailin Wang

**Affiliations:** ^1^ The State Key Laboratory of Environmental Chemistry and Ecotoxicology, Research Center for Eco-Environmental Sciences, Chinese Academy of Sciences, Beijing 100085, China; ^2^ University of Chinese Academy of Sciences, Beijing 100039, China

**Keywords:** vitamin C, PD0325901, hypomethylation, embryonic stem cells, Prdm14

## Abstract

A rationally selected combination of small-molecule chemicals can affect cell plasticity and fate, suggesting an open chemistry way to manipulate cells to achieve a specific goal. Here we for the first time demonstrate that a combination of vitamin C (Vc) and PD0325901 can achieve about 90% erasure of 5-methylcytosine (5mC) within 5 days (decreasing from 3.2 to ~ 0.3 5mC per 100 C) in mouse embryonic stem cells (ESCs). The hypomethylated level is comparable to that of gonadal primordial germ cells (PGCs), whose pluripotency is closely associated with the global DNA hypomethylation. In contrast, Vc or PD0325901 alone only induces a moderately reduced level of global DNA methylation. Our mechanistic study suggested that PD0325901 elevated expression of Prdm14, which repressed de novo methyltransferase Dnmt3b and its cofactor Dnmt3l at levels of protein, via the mode to eliminate 5mC from de novo synthesis. By further addition of Vc, the oxidation of 5mC as catalyzed by Tet1/Tet2 dioxygenases was significantly increased as manifested by the elevated level of 5-hydroxymethylcytosine. However, by the depletion of Tet1/Tet2, Vc failed to enhance PD0325901-stimulated hypomethylation of ESCs’ genomic DNA. Furthermore, mouse ESCs in Vc/PD0325901-supplemented medium show great morphology and pluripotency. Therefore, we demonstrate a novel and synergistic chemical approach for promoting hypomethylation and sustaining pluripotency of ESCs.

## INTRODUCTION

Mammalian cells demonstrate an amazing plasticity that allows them to develop from one type to the other functionally distinct cells. Embryonic stem cells (ESCs) are just such cells. ESCs are derived from the inner cell mass (ICM) of a developing blastocyst [1]. These cells are in a pluripotent state and are capable of forming all somatic cell lineages and germ cells [2]. Establishment of ESCs can provide the chance to investigate the developmental processes *in vitro* [3]. Furthermore, due to their pluripotency, various cell types can be generated for regenerative medicine [3].

5mC is the product of the methylation of cytosine at carbon-5 position, which is catalyzed by DNA methyltransferases, DNMT1, DNMT3a and DNMT3b, in mammalian cells. In somatic cells, 5mC (combined with other proteins) functions in regulation of gene expression, X-chromosome inactivation and gene imprinting [4–7]. Interestingly, global erasure of genomic 5mC occurs in ESCs in blastocysts [8], and the hypomethylation in ESCs is closely linked with pluripotent state [9]. However, unlike ESCs in blastocysts, serum-cultured ES cells often exhibit global hypermethylation [9].

Many studies have shown that a combination of small-molecule chemicals can guide cell fates. As manifested by recent discovery of full chemically induced pluripotent stem cells, neural progenitor cells, and cardiomyocytes [10–12], small-molecule compounds show promising applications in reprogramming and trans-differentiation and the potentials in clinical developments.

Here, we present a chemical approach to rapidly and effectively promote hypomethylation in mouse embryonic stem cells (ESCs) using two small-molecule compounds. It is known that both vitamin C [13, 14] and 2i (two small-molecule kinase inhibitors, PD0325901 and CHIR99021) [9, 15] can induce a decrease in genomic 5mC, respectively. Interestingly, they exert 5mC erasure through two distinct mechanisms. Vc enhances demethylation activity, while 2i repress de novo DNA methylation synthesis. It is reported that the effects of Vc on DNA methylation are greater in 2i medium relative to that in serum at several gene promoters, however, the mechanism is not unclarified [14]. Here, we attempted to explore a synergistic effect of Vc and 2i on the erasure of genomic 5mC in mouse ESCs grown in FBS medium. Furthermore, we will investigate which factor in 2i contributes to the combined DNA demethylation and unveil the mechanisms of action.

## RESULTS AND DISCUSSION

### 2i and Vc synergistically induced more pronounced DNA hypomethylation of mouse ESCs

**Figure 1:**
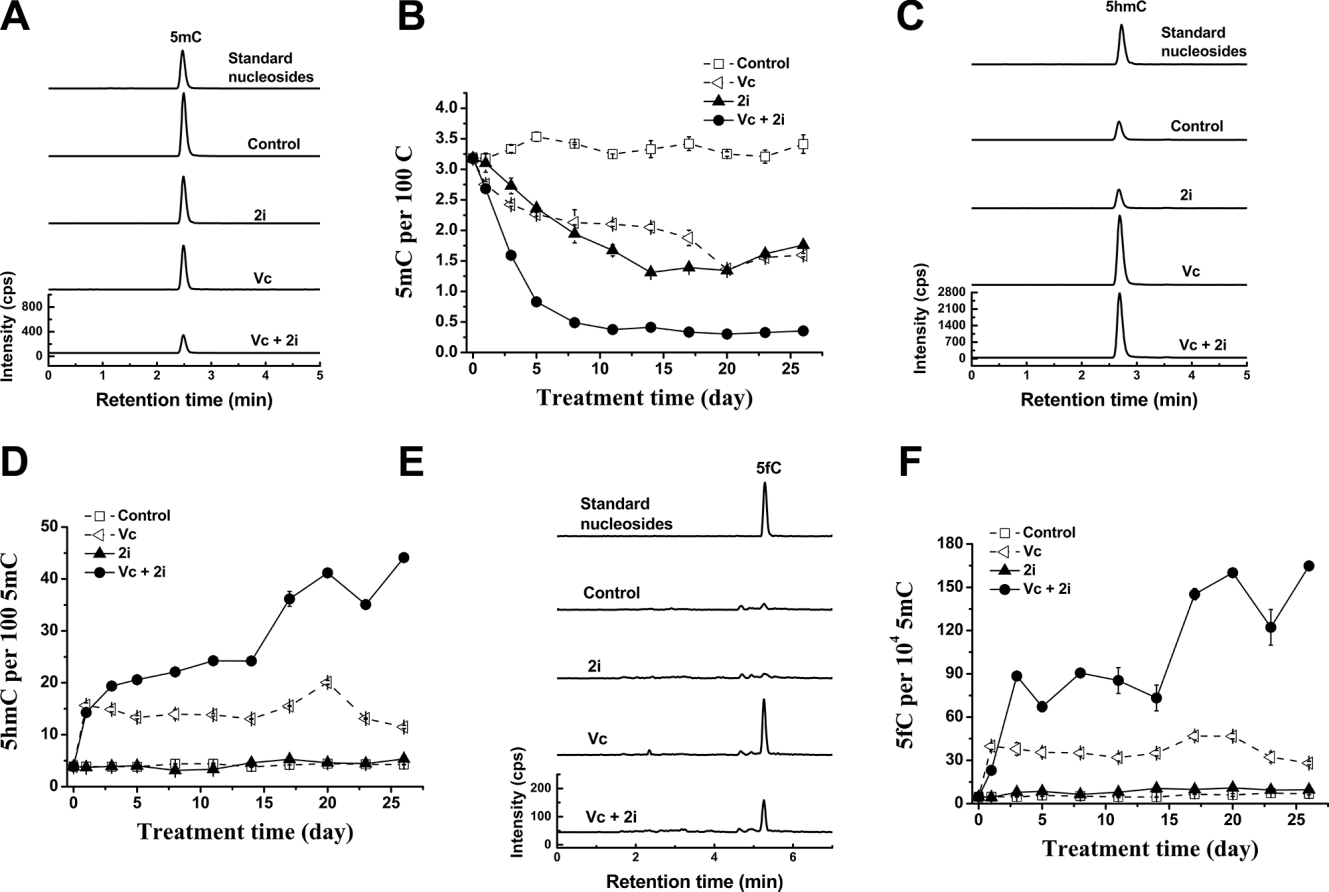
Vc/2i synergistically induced DNA hypomethylation of mouse ESCs UHPLC-MS/MS detection of 5mC at 5 day (**A**), 5hmC (**C**) and 5fC (**E**) at 1 day after addition of Vc and 2i singly or in combination. 5mC (5mC/C, **B**), 5hmC (5hmC/5mC, **D**) and 5fC (5fC/5mC, **F**) frequency time-dependent alteration during 26-day culture of Vc and 2i alone or simultaneously.

By the use of highly sensitive approach of ultrahigh performance liquid chromatography-triple quadrupole mass spectrometry coupled with multiple-reaction monitoring (UHPLC-MRM-MS/MS) [13], we observed that the co-treatment of Vc and 2i can dramatically reduce 5mC content of mouse ESCs. After 11 days-treatment, the level of 5mC was sustained at 0.33 ± 0.01 per 100 C, indicating a loss of about 90% 5mC or a retention of 10% 5mC (Figure 1A and 1B). In contrast, without treatment, 5mC content slightly waved around 3.3 ± 0.14 per 100 C during the cultivation period of 26 days (Figure 1B). With an addition of 2i or Vc to the basic medium, 5mC level declined to about 1.3 ± 0.02 per 100 C (~61% reduction) or 1.4 ± 0.01 per 100 C (~ 58% decrease). The results that either 2i or Vc induced hypomethylation of mouse ESCs were consistent with previous work [9, 13–15].

We speculated that the synergistic hypomethylation of mouse ESCs induced by co-treatment of 2i and Vc may be partly related to enhanced DNA demethylation activity. Therefore, we examined genomic 5mC oxidation products, which function as the intermediates of DNA demethylation. It is known that 5mC can be oxidized to produce 5-hydroxymethylcytosine (5hmC), which is catalyzed by Tet family dioxygenases in a Fe (II) and 2-oxoglutarate-dependent manner [16, 17]. 5hmC in genomic DNA may undergo replication-dependent loss [18]. Alternatively, 5hmC can be further oxidized to 5-formylcytosine (5fC) and 5-carboxylcytosine (5caC). The latter two bases in genomic DNA can be efficiently excised by thymine DNA glycosylase (TDG) to regenerate unmethylated cytosine, suggesting an active DNA demethylation [19, 20].

As shown in Figure 1C–1F, accompanying with the reduction in 5mC, we also observed that the levels of 5hmC (Figure 1C and 1D) and its iterative oxidation product 5fC (Figure 1E and 1F) increased upon Vc treatment or Vc/2i co-treatment. This is consistent with our previous work showing that Vc can enhance Tet activity and promote the generation of 5hmC [13]. In comparison with Vc treatment, co-treatment of Vc and 2i can further increase the frequency of 5hmC (44 in per 100 5mC at 26 day) (Figure 1D). We also observed that relative to Vc treatment alone, the 5fC frequency further increased when the mouse ESCs were co-treated with Vc and 2i (165 in per 10^4^ 5mC at 26 day) (Figure 1F). Taken together, these data support that Vc and 2i synergistically drove the faster erasure of DNA methylation, partly relating to the enhanced DNA demethylation activity.

### Vc/PD0325901 caused demethylation at faster kinetics than Vc/2i

Next question is which component of 2i (a combination of PD0325901 and CHIR99021) [21] contributes to the global loss of 5mC in genomic DNA of mouse ESCs. To answer this question, we tested the combination of Vc with either PD0325901 (PD) or CHIR99021 (CH).

**Figure 2:**
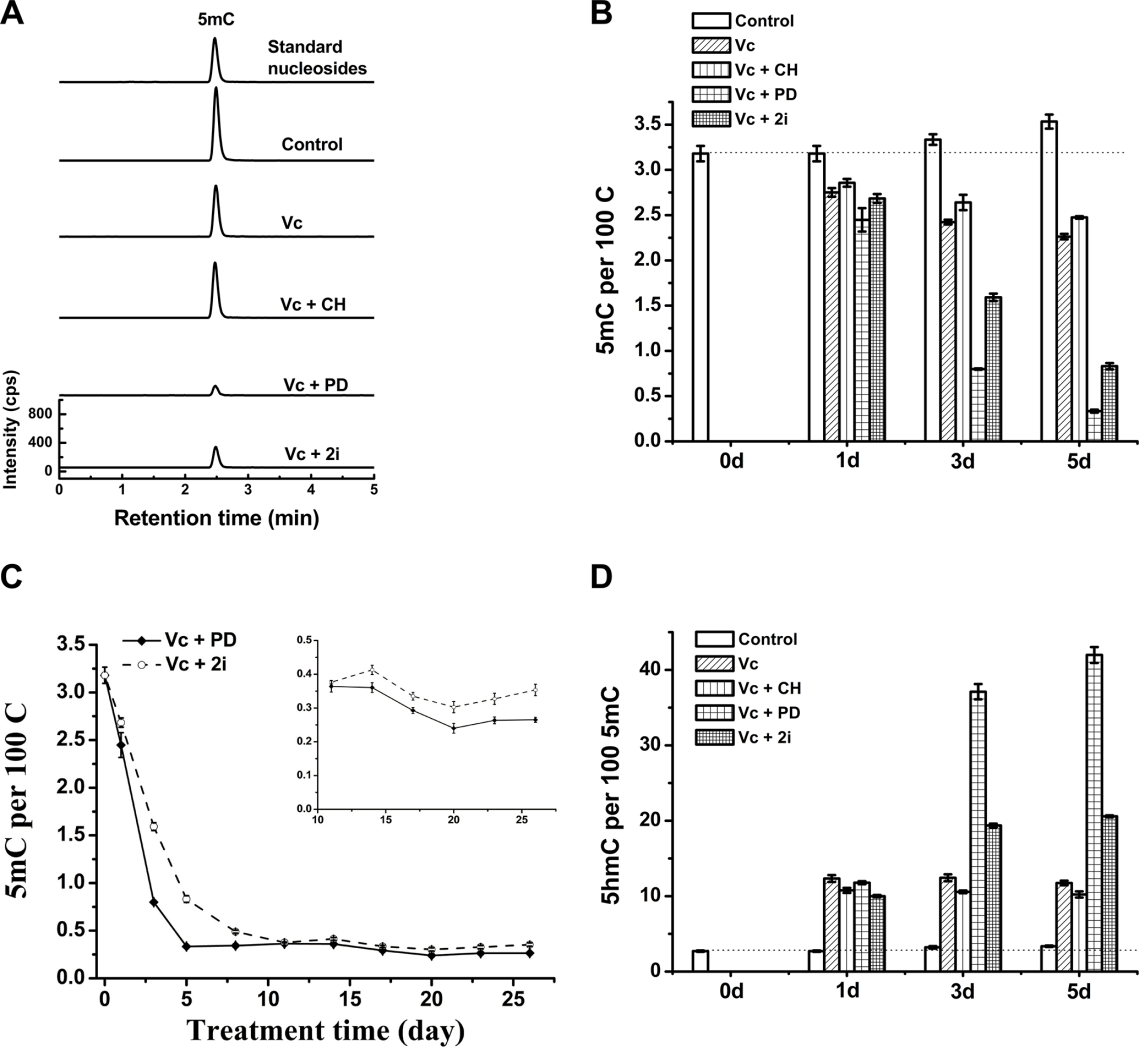
Vc/PD0325901 led to more striking loss of DNA methylation than Vc/2i UHPLC-MS/MS analysis of 5mC of mouse ESCs at 5 day (**A**) and 5mC time-dependent change within 5 days (**B**) after treatment with Vc, Vc/PD0325901, Vc/CHIR99021 and Vc/2i, respectively. (**C**) Comparison of decline kinetics in 5mC between Vc/PD0325901 and Vc/2i during 26 days of treatment. The insert showed the variation trend of 5mC after 11 days. (**D**) 5hmC frequency (5hmC/5mC) change within 5 days after Vc co-treatment with PD0325901 or CHIR99021 or 2i.

As shown by Figure 2A and 2B, similar to the co-treatment of Vc and 2i, the co-treatment of PD0325901 and Vc can induce global and dramatic reduction of genomic 5mC, but not the co-treatment of Vc and CHIR99021. Interestingly, addition of CHIR99021 slightly suppressed Vc-stimulated demethylation (Figure 2B), whereas Vc/PD0325901 treatment exhibited faster kinetics of 5mC loss than Vc/2i co-treatment during 26-day culture of ESCs (Figure 2C). At 11 day, 5mC level was comparable between Vc/PD0325901 and Vc/2i treatment. The insert in Figure 2C showed the variation tendency of 5mC after 11 days. The obtained minimum levels of genomic 5mC are 0.33 ± 0.01 per 100 C and 0.26 ± 0.01 per 100 C for the co-treatment of Vc/2i and Vc/PD0325901, respectively. Consistently, Vc/PD0325901 also showed a higher 5hmC frequency (42 in per 100 5mC at 5 day, Figure 2D), due to the combination of Vc-promoted 5hmC increase and Vc/PD0325901-induced loss of 5mC. These results clearly prove that PD0325901 but not CHIR99021 in two small-molecule kinase inhibitors (2i) contributes to reduced level of genomic 5mC in ESCs. Regarding the reason that Vc/PD0325901 drove a faster 5mC loss than Vc/2i, we suspect that it is due to slight inhibition of CHIR99021 on demethylation. Since CHIR99021 can inhibit pluripotency-related GSK3β pathway [21], this also suggests that currently and previously observed 2i-induced hypomethylation of ESCs is not pertinent to the inhibition of GSK3β pathway.

Essentially, the Vc/PD0325901 co-treatment induces a loss of 92.1% genomic 5mC. In other words, only 7.9% genomic 5mC (0.26 ± 0.01 per 100 C) is resistant to the enhanced 5mC erasure (Figure 2C). The question is that which genes are retained in Vc/PD0325901-induced erasure of methylation. Studies have shown that imprinted genes, intracisternal A particles and major satellites remained relatively resistant to 2i-induced loss of 5mC [9]. Similarly, imprinted regions and intracisternal A particle retroelements were also resistant to vitamin C-induced demethylation in N2B27-based 2i medium [14]. Probably these imprinted regions and intracisternal A particles are also resistant to Vc/PD0325901-induced erasure of methylation. However, to exactly identify the resistant genes will require further genome-wide sequencing of 5mC and 5hmC.

### Tet-dependent DNA demethylation promoted by vitamin C contributed to global hypomethylation induced by PD0325901/Vc

**Figure 3:**
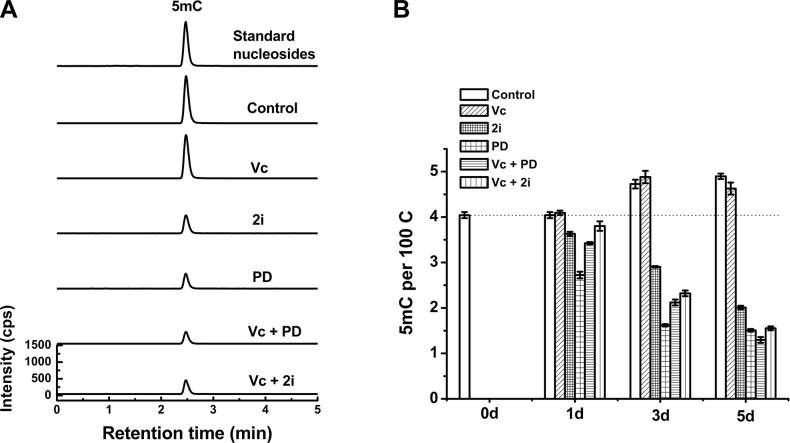
Tet1/Tet2 double knockout attenuated the loss of 5mC induced by Vc/PD0325901 and Vc/2i relative to that in wild-type ESCs UHPLC-MS/MS analysis of 5mC at 5 day (**A**) and measured the alteration of 5mC within 5 days (**B**) after treatment with Vc, 2i, PD0325901, Vc/PD0325901 and Vc/2i.

We next asked whether Tet family dioxygenases contributed to the synergistic hypomethylated state caused by Vc/PD0325901 co-treatment. Regarding the fact that Tet1 and Tet2 are two main Tet enzymes expressed in ESCs, we investigated the 5mC erasure in Tet1/Tet2 depleted ESCs. As shown in Figure 3A and 3B, the depletion of Tet1/Tet2 abolished the effect of Vc on DNA methylation. In contrast, by the double knockout of Tet1/Tet2, the treatment of 2i or PD0325901 can still reduce 5mC level progressively within 5 days due to a Tet-independent demethylation mechanism. Furthermore, we found that PD0325901 can trigger more pronounced loss of 5mC compared to 2i, which explain the discrepancy of Vc/PD0325901 and Vc/2i-induced erasure of 5mC in wild-type ESCs. However, co-treated by Vc/2i or Vc/PD0325901 did not lead to a more striking loss in 5mC relative to 2i or PD0325901 alone upon deleting Tet1/Tet2. The levels of 5mC at 5 day are comparable between 2i ± Vc and PD0325901 ± Vc, which are down to 1.5–2.0 5mC per 100C. Moreover, after 5-day co-treatment of Vc/PD0325901, the level of global 5mC in Tet1/Tet2 knockout ESCs (1.3 per 100 C, Figure 3) is much higher than that of the wild-type of ESCs (0.33 per 100 C, Figure 2B). These results support that the depletion of Tet1/Tet2 not only abolished the demethylation effect of Vc, and also eliminated the synergistic 5mC erasure caused by the co-treatments of Vc/2i and Vc/PD0325901. Collectively, vitamin C-enhanced Tet1/Tet2 activity contributes to the synergistic demethylation of Vc/2i and Vc/PD0325901.

### PD0325901 upregulated Prdm14, accompanying with downregulation of Dnmt3b and Dnmt3l, but not Dnmt3a

**Figure 4:**
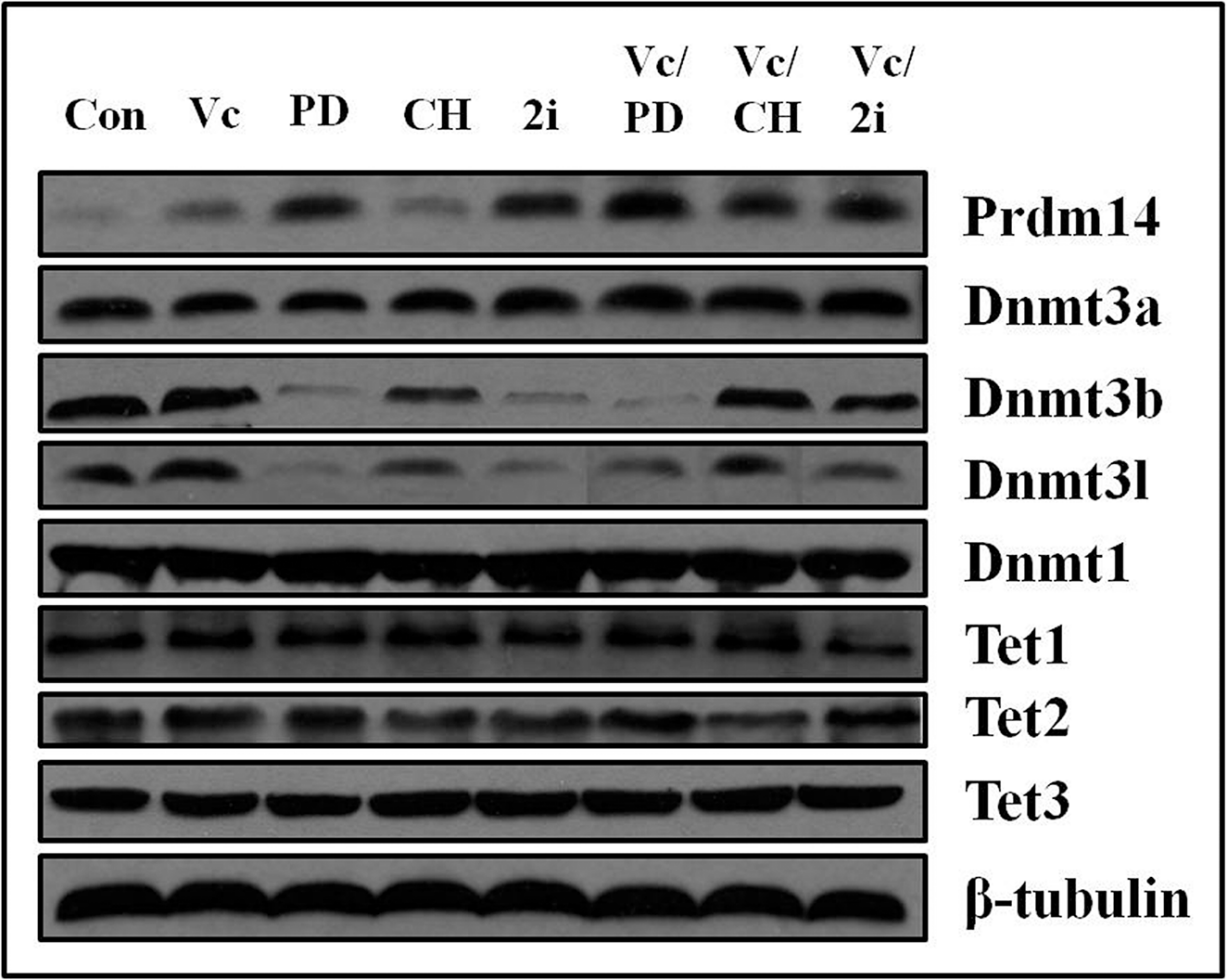
The expression of Prdm14, Dnmt3b and Dnmt3l was altered by PD0325901-containing treatment and Dnmt3a, Dnmt1, Tet1, Tet2 and Tet3 exhibited no change in various culture conditions β-tubulin was used as the internal reference. The bands from left to right corresponded to the treatment of control, Vc, PD0325901, CHIR99021, 2i, Vc/PD0325901, Vc/CHIR99021 and Vc/2i, respectively.

**Figure 5:**
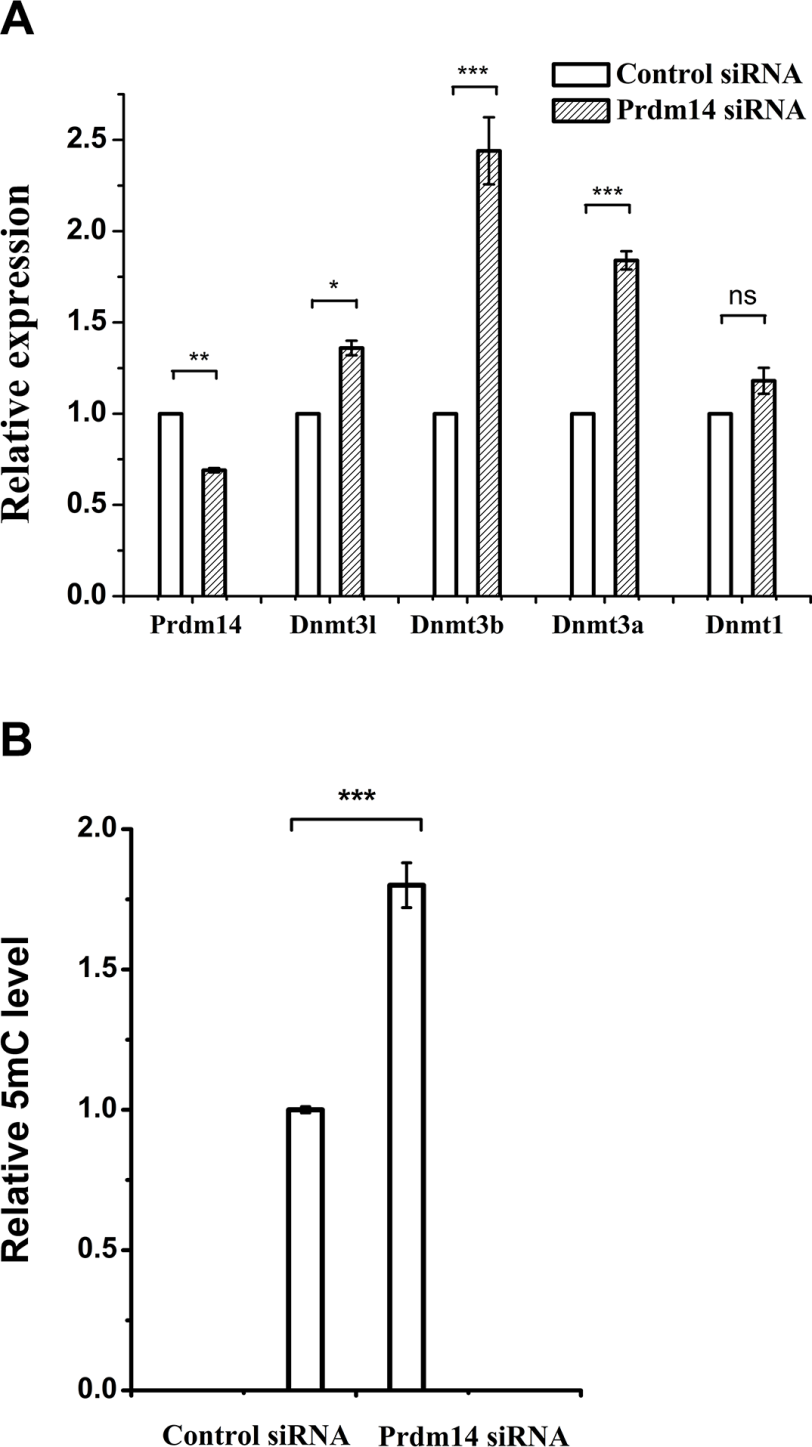
The effect of Prdm14 on Dnmt and DNA methylation in mouse ESCs (**A**) qPCR analysis of Prdm14 and Dnmt mRNA expression levels after the downregulation of Prdm14 in comparison with control siRNA. Error bars show s.d. Unpaired student's *t*-test was used to assess the statistical significance. *P < 0.05, **P < 0.01, ***P < 0.001, ns represents no statistical significance. (**B**) UHPLC-MS/MS analysis of global 5mC levels after knockdown of Prdm14 compared to control siRNA.

Since PD0325901 cannot induce 5mC erasure through Tet-mediated demethylation, we further examined the protein expression of DNA methyltransferases (Dnmt1, Dnmt3a, and Dnmt3b) and related regulation genes (Dnmt3l and Prdm14) [9, 22, 23]. As shown by western blot analysis, Prdm14 was considerably up-regulated only in PD0325901-containing treatment (Figure 4). Meanwhile, Dnmt3b and its cofactor Dnmt3l, were strongly down-regulated in PD0325901-containing treatment. However, the expression level of Dnmt3a, another de novo DNA methyltransferase, was not reduced. The reason is not clear. The maintenance DNA methyltransferase Dnmt1 also showed no change. Additionally, Tet1, Tet2 and Tet3, the known three DNA demethylation-related enzymes, were not altered significantly at the protein expression levels (Figure 4). Recent studies have suggested that Prdm14 repressed the expression of de novo DNA methyltransferases Dnmt3a, Dnmt3b and their cofactor Dnmt3l by recruiting polycomb repressive complex 2 (PRC2) to their promoters and played a key role in maintaining hypomethylation in 2i conditions [9, 22, 24]. We further confirm the relevance between Prdm14 and loss of methylation in our Vc/PD0325901 system. Initially, we culture mouse ESCs in basic medium supplemented with Vc/PD0325901 for 5 days to elevate the expression of Prdm14. Next, we downregulate the levels of Prdm14 with siRNA to examine the alteration of Dnmt and 5mC level. We observed by qPCR that mRNA expression of Prdm14 decreased 31% after silencing. Following by the downregulation of Prdm14, Dnmt3a and Dnmt3b were elevated by 1.84-fold (*p* < 0.001) and 2.44-fold (*p* < 0.001), respectively, and their regulator Dnmt3l was also upregulated by 1.36-fold (Figure 5A). In contrast, the mRNA level of Dnmt1 kept constant. Accompanying with the downregulation of Prdm14, 5mC content increased by 1.8-fold (Figure 5B). Collectively, these data suggested that PD0325901 promotes the expression of Prdm14, which further down-regulates Dnmt3b and Dnmt3l. By the mechanism, de novo synthesis of genomic 5mC is greatly reduced.

### The effect of Vc/PD0325901 on pluripotency of mouse ESCs

**Figure 6:**
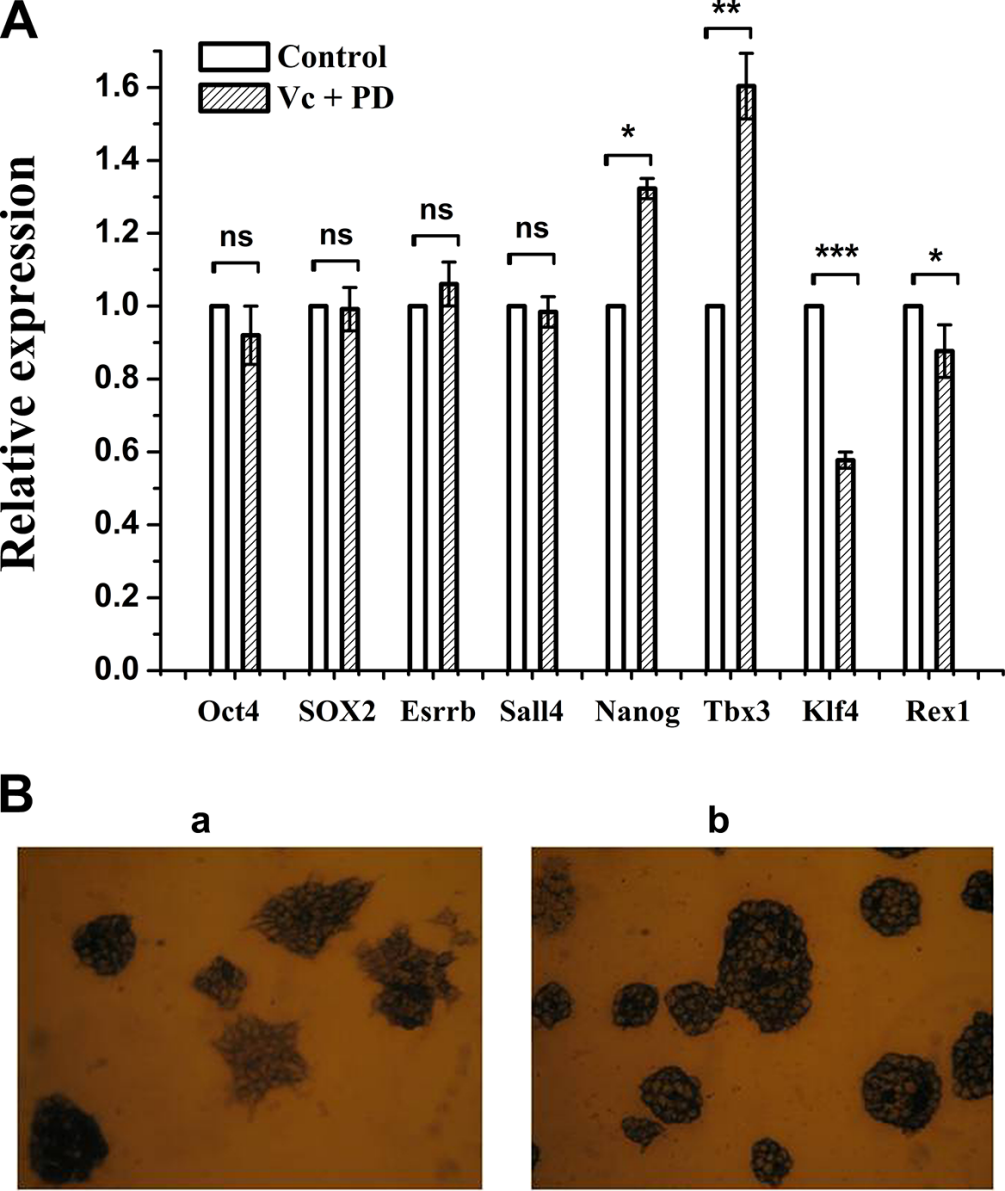
The effect of Vc/PD0325901 on pluripotency-related genes and alkaline phosphatase (**A**) Expression of partial pluripotency genes was changed at 5 day of Vc/PD0325901 treatment relative to FBS untreated sample. Data were represented as mean ± s.d. (**B**) Mouse ESCs maintained in Vc/PD0325901-supplemented medium showed great morphology and an undifferentiated state (b) relative to ESCs grown in FBS (a) at 26 day.

As we show, co-treatment of Vc and PD0325901 can induce global hypomethylation of mouse ESCs within 5 days, however, it is not known whether pluripotency can be maintained under these conditions. To answer this question, we tested mRNA expression of several core pluripotency factors which have been functionally validated as playing vital roles [25]. The results revealed that expression levels of Oct4, SOX2, Esrrb and Sall4 remained constant after 5 days of Vc/PD0325901 treatment relative to culture in serum (Figure 6A). Nanog and Tcf3 showed 1.3-fold and 1.6-fold increase, respectively, while Klf4 was observed by 42% decrease and Rex1 only exhibited about 12% downregulation. The results indicated pluripotency factors showed diverse alteration of expression in Vc/PD0325901 co-treatment, however, the expression of most core factors, Oct4 and SOX2, was relatively uniform. Recent studies have suggested that pluripotency genes expression is not changed by Vc, whereas 2i increases expression of Nanog [14]. Therefore, alteration of pluripotency genes expression induced by Vc/PD0325901 should mainly be attributed to the action of PD0325901.

We next examine the pluripotency using alkaline phosphatase (AP) assay. Through AP staining assay, we can monitor ESCs undifferentiation or differentiation. After continuous culture of mouse ESCs in Vc/PD0325901-supplemented medium for 26 days, as shown in Figure 6B (b), we observed that ESCs were well stained and ball-like without outgrowth, that is, cells remained an undifferentiated state. In contrast, ESCs grown in serum for 26 days showed the purple-black and ball-like state in part, while others exhibited light color with spread (Figure 6B (a)), indicating that ESCs in serum showed heterogeneity in morphology and pluripotency, in line with other reports [15]. The results revealed that PD0325901 together with Vc can maintain ES cells in great morphology and an undifferentiated state.

In summary, we demonstrated a synergistic mechanism of enhancing DNA demethylation and repressing DNA methylation simultaneously in ESCs and developed a novel approach for maintaining ES cells at a hypomethylated and undifferentiated state and in great morphology.

## MATERIALS AND METHODS

### Cell culture conditions and treatment

Mouse embryonic stem cells of wild type (WT mouse ESCs, 129 SvEv) and of Tet1/Tet2 double knock-out (Tet1/Tet2^−/−^) were employed in the assay. Initially, cells were maintained in high-glucose Dulbecco's Modified Eagle Medium (DMEM) (HyClone) supplemented with 20% ES FBS (Corning), 1000 units/ml leukemia inhibitory factor (LIF) (Millipore), 0.1 mM non-essential amino acids (NEAA), 1 mM sodium pyruvate (Gibco), 2 mM L-glutamine, 0.1 mM β-mercaptoethanol, 33 IU/ml penicillin and 33 μg/ml streptomycin (Corning). We define the medium described above as the basic medium which may be added extra small molecule. For cultures without feeders, cells were grown in 0.1% gelatin-coated dishes and were incubated at 37°C in a humid atmosphere with 5% CO_2_.

To investigate the effect of small-molecule compounds on the DNA methylation, the basic medium was supplemented with 1.0 μM PD0325901 (Stemolecule), 3.0 μM CHIR99021 (Stemolecule) and 100 μM vitamin C alone or in combination. When cells grew to 50% confluence, fresh medium with small-molecule compounds was used for additional culture for 24 h or longer. When the exposure time was reached, cells were dissociated with trypsin and then were split into two parts, nine tenths for DNA extraction and another one tenth for further culture in the corresponding culture medium which was changed daily.

### DNA extraction and enzymatic digestion

Genomic DNA extraction was performed with a Genomic DNA Purification Kit (Promega) according to the manufacturer's instructions. The concentration of extracted DNA was determined with NanoDrop 2000 (Thermo Scientific) and quality was evaluated with the ratio of absorbance at 260 nm and 280 nm.

To obtain mononucleosides, 5 μg DNA was digested with 2 U calf intestinal phosphatase, 1 U DNase I and 0.005 U snake venom phosphodiesterase I (New England Biolabs) at 37°C overnight. To remove enzymes used for digestion, DNA solution was filtered with ultra-filtration tubes with molecule weight cutoff at 3 KDa (Pall). The filter solution was analyzed with UHPLC-MS/MS for 5mC, 5hmC and 5fC.

### UHPLC-MS/MS analysis of 5mC, 5hmC and 5fC

The UHPLC-MS/MS analysis was performed according to the published methods [13] with a minor modification. The Agilent 1290 UHPLC system and Zorbax Eclipse Plus C18 column (100 mm × 2.1 mm, 1.8 μm, Agilent Technologies) were adopted for 5mC, 5hmC and 5fC analysis. In brief, the mobile phase consisting of 5.0% methanol and 95% water with 0.1% formic acid was applied for 5mC analysis. An optimized gradient elution was used for 5hmC and 5fC analysis: 0–3 min, 5.0% B; 3–6min, 15.0% B; 6–10 min, 100% B; 10–15 min, 5.0% B. Solvent A was 2.0 mM NH_4_HCO_3_ aqueous solution (pH 9.0), and solvent B was 100% methanol. The flow rate was set at 0.25 mL/min. The elution from the column was directly injected into ESI-G6410B triple quadrupole mass spectrometer. The multiple reaction monitoring (MRM) mode was employed for the experiment, and set as follows: m/z 242→126 for 5mC (collision energy, 5eV); m/z 228→112 for dC (5eV); m/z 258→142 for 5hmC (5eV) and m/z 256→140 for 5fC (5eV). The capillary and fragment voltages were set at +3500 V and 90 V, respectively. Each sample was analyzed three times with an injection volume of 5 μl. The stable isotopes 5′-(methyl-d_3_)-2′-deoxycytidine ([D_3_]) 5mC (Torto Research Chemicals), 5′-(hydroxymethyl-d_3_)-2′-deoxycytidine ([D_3_]) 5hmC and [^15^N_3_]-dC (Cambridge Isotope laboratories, Inc.) were used as the internal standard for the calibration of 5mC, 5hmC and 5fC. The 5mC, 5hmC and 5fC concentrations were estimated based on the corresponding standard curves.

### Western blot analysis

Whole cell protein extracts were prepared with cold RIPA buffer. Total proteins (50 μg) were separated on SDS-PAGE and then transferred to polyvinylidene fluoride (PVDF) membranes. After blocking in 5% non-fat milk, membranes were incubated with the indicated primary antibody and secondary antibodies before visualization Primary antibodies used were DNMT1 (abcam, ab13537, 1:1000), DNMT3a (bioworld, BS6587, 1:1000), DNMT3b (abcam, ab13604, 1:500), DNMT3l (abcam, ab3493, 1:1000), Prdm14 (bioworld, BS7634, 1:1000), Tet1 (bioworld, BS6966, 1:1000), Tet2 (bioworld, BS7804, 1:1000), Tet3 (abcam, ab135033, 1:700) and β-tubulin (bioworld, BS1482MH, 1:1500). Secondary antibodies used were goat anti-rabbit IgG (1:5000) and goat anti-mouse IgG (1:5000).

### Gene silencing and real-time quantitative PCR

Prdm14 mRNA in WT mouse ESCs was silenced by siRNAs purchased from GenePharma. SiRNA transfection was performed by Lipofectamine RNAi MAX according to the manufacturer's instructions and the effect of RNAi was examined after treatment for 3 days. To ensure the efficiency of RNAi, a second transfection was carried out after transfection for 1 day.

After 3 days of transfection, total RNA was extracted from cells with Trizol reagent (Life technologies Corporation). 1 μg RNA was reversely transcribed to cDNA with Promega reverse transcription system. Real-time PCR was performed using Promega GoTaq qPCR Master Mix following the manufacturer's protocol on Stratagene Mx3005P real-time PCR System (Aglient Technologies). The mRNA levels of 5mC-related and pluripotent genes were examined and the relative expression of genes was normalized to the housekeeper gene Gapdh. Two-tailed and unpaired *t*-tests were performed with Graphpad Prism 5 software. Primer sequences were listed as below.

Prdm14 forward: 5′-CAGCGACTTCATTGCCAAA GGAG-3′

Prdm14 reverse: 5′-GCCGTCGATAAAATGGCT CAGG-3′

Dnmt3a forward: 5′-CGCAAAGCCATCTACGAA GTCC-3′

Dnmt3a reverse: 5′- GCTTGTTCTGCACTTCCA CAGC-3′

Dnmt3b forward: 5′-CGCACAACCAATGACTCT GCTG-3′

Dnmt3b reverse: 5′-GGTGACTTCAGAAGCCAT CCGT-3′

Dnmt1 forward: 5′-GGACAAGGAGAATGCCATG AAGC-3′

Dnmt1 reverse: 5′-TTACTCCGTCCAGTGCCA CCAA-3′

Dnmt3l forward: 5′-CTGTGGAACTCTCCAGGT GTAC-3′

Dnmt3l reverse: 5′- GTGCAGTAACTCTGGTGTC CATC-3′

Gapdh forward: 5′-GTGTTCCTACCCCCAATG TGT-3′

Gapdh reverse: 5′-ATTGTCATACCAGGAAAT GAGCTT-3′

Oct4 forward: 5′-AGTCTGGAGACCATGTTTCT GAAGT-3′

Oct4 reverse: 5′-TACTCTTCTCGTTGGGAATA CTCAATA-3′

SOX2 forward: 5′-CATGAGAGCAAGTACTGG CAAG-3′

SOX2 reverse: 5′-CCAACGATATCAACCTGC ATGG-3′

Nanog forward: 5′-AGGACAGGTTTCAGAAGCA GAAGT-3′

Nanog reverse: 5′-TCAGACCATTGCTAGTCTTC AACC-3′

Klf4 forward: 5′-AGGAGCCCAAGCCAAAG AGG-3′

Klf4 reverse: 5′-CGCAGGTGTGCCTTGAG ATG-3′

Esrrb forward: 5′-CAGGCAAGGATGACAGA CG-3′

Esrrb reverse: 5′-GAGACAGCACGAAGGACT GC-3′

Rex1 forward: 5′-GAGACTGAGGAAGATGGCT TCC-3′

Rex1 reverse: 5′-CTGGCGAGAAAGGTTTTGC TCC-3′

Sall4 forward: 5′-GTTAGATGTCAAGGCCAAGG AC-3′

Sall4 reverse: 5′-GGCGTCTACAGAGAGACTC GAT-3′

Tbx3 forward: 5′-TTATTTCCAGGTCAGGAGATG GC-3′

Tbx3 reverse: 5′-GGTCGTTTGAACCAAGTCCC TC-3′

### Alkaline phosphatase staining

Alkaline phosphatase staining was performed by StemTAG™ alkaline phosphatase staining and activity assay kit (colorimetric) (Cells Biolabs, Inc.) according to the manufacturer's instructions.
